# Antioxidant and Neuroprotective Activity of Vitamin E Homologues: In Vitro Study

**DOI:** 10.3390/metabo12070608

**Published:** 2022-06-30

**Authors:** Agnieszka Trela-Makowej, Monika Leśkiewicz, Jerzy Kruk, Andrzej Żądło, Agnieszka Basta-Kaim, Renata Szymańska

**Affiliations:** 1 Faculty of Physics and Applied Computer Science, AGH University of Science and Technology, Reymonta 19, 30-059 Cracow, Poland; agnieszka.trela@fis.agh.edu.pl; 2 Department of Experimental Neuroendocrinology, Maj Institute of Pharmacology, Polish Academy of Sciences, Smętna 12, 31-343 Cracow, Poland; leskiew@if-pan.krakow.pl (M.L.); basta@if-pan.krakow.pl (A.B.-K.); 3 Department of Plant Physiology and Biochemistry, Faculty of Biochemistry, Biophysics and Biotechnology, Jagiellonian University, Gronostajowa 7, 30-387 Cracow, Poland; jerzy.kruk@uj.edu.pl; 4 Department of Biophysics, Faculty of Biochemistry, Biophysics and Biotechnology, Jagiellonian University, Gronostajowa 7, 30-387 Cracow, Poland; andrzej.zadlo@uj.edu.pl; 5 Department of Biophysics, Jagiellonian University Medical College, św. Łazarza 16, 31-530 Cracow, Poland

**Keywords:** antioxidants, liposomes, tocochromanols, lipid peroxidation, lipid membrane, neuroprotection, neuroblastoma cell line

## Abstract

Here we present comparative data on the inhibition of lipid peroxidation by a variety of tocochromanols in liposomes. We also show for the first time the potential neuroprotective role of all the vitamin E homologues investigated on the neuronally differentiated human neuroblastoma SH-SY5Y cell line. α-Tocopherol had nearly no effect in the inhibition of lipid peroxidation, while β-, γ-, and δ-tocopherols inhibited the reaction completely when it was initiated in a lipid phase. Similar effects were observed for tocotrienol homologues. Moreover, in this respect plastochromanol-8 was as effective as β-, γ-, and δ-tocochromanols. When the prenyllipids were investigated in a 1,1-diphenyl-2-picrylhydrazyl (DPPH) test and incorporated into different lipid carriers, the radical oxidation was most pronounced in liposomes, followed by mixed micelles and the micellar system. When the reaction of tocochromanols was examined in niosomes, the oxidation was most pronounced for α-tocopherol and plastochromanol-8, followed by α-tocotrienol. Next, using retinoic acid-differentiated SH-SY5Y cells, we tested the protective effects of the compounds investigated on hydrogen peroxide (H_2_O_2_)-induced cell damage. We showed that tocotrienols were more active than tocopherols in the oxidative stress model. Plastochromanol-8 had a strong inhibitory effect on H_2_O_2_-induced lactate dehydrogenase (LDH) release and H_2_O_2_-induced decrease in cell viability. The water-soluble α-tocopherol phosphate had neuroprotective effects at all the concentrations analyzed. The results clearly indicate that structural differences between vitamin E homologues reflect their different biological activity and indicate their potential application in pharmacological treatments for neurodegenerative diseases. In this respect, the application of optimal tocochromanol-carrying structures might be critical.

## 1. Introduction

**Figure 1:**
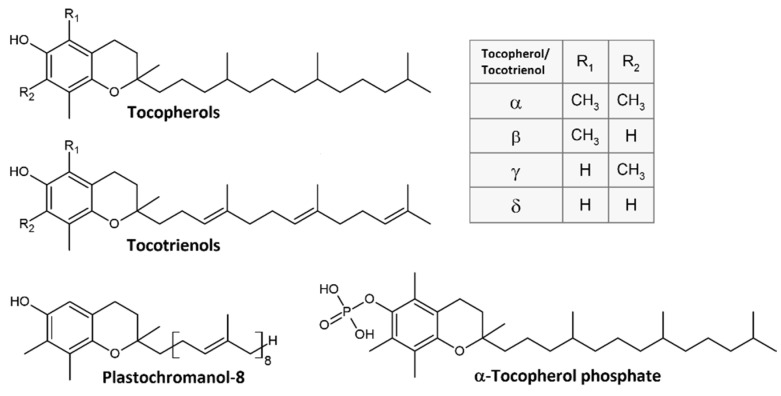
Chemical structure of tocochromanols.

Vitamin E includes a complex of naturally occurring tocochromanols [1,2,3,4], such as tocopherols (Toc), tocotrienols (Tt), and their derivatives [5,6,7,8,9,10,11,12]. The number and position of methyl groups in the chromanol ring define four naturally occurring α-, β-, γ-, and δ-Toc and Tt homologues [1,13]. Plastochromanol-8 (PC-8) is a γ-Tt homologue with a longer side chain [14,15] (Figure 1). Moreover, the occurrence of a water-soluble α-tocopherol phosphate (α-TP) has been reported in biological systems [16]. Apart from α-TP, all the other tocochromanols are lipid-soluble antioxidants, which play a pivotal role in human health and nutrition [3]. They are generally found at high concentrations in vegetable oils such as almond, safflower, canola oil, or in other high-fat sources such as nuts, seeds, or grains [17]. The growing consumption of plant-derived oils rich in polyunsaturated fatty acids (PUFAs) that are absorbed into membrane phospholipids highlights the role of tocochromanols in antioxidant protection.

α-Toc, as a synonym of vitamin E for humans, is the most frequently examined tocopherol form, but many recent studies have indicated that other forms of vitamin E may exhibit more potent biological activity [18,19], including their protective role in the prevention or treatment of chronic cardiovascular diseases, immune system disorders, and cancer [20,21,22,23,24]. Emerging evidence suggests that vitamin E homologues (non-α-Toc) and metabolites have strong anti-inflammatory effects [25,26]. It has been observed that vitamin E deficiency increases the risk of dementia and other neurological disorders, such as Alzheimer’s or Parkinson’s disease. This effect has been shown in several epidemiological studies [27,28,29]. Moreover, the supplementation of vitamin E (α-Toc) could reverse the neurologic dysfunction [23,30]. In humans and various animal models, the results are contradictory. Some data strongly indicate that α-Tt protects against neurodegeneration [24,30]. In contrast, several clinical trials have shown that vitamin E (α-Toc alone or as a mixture of Tocs in plant oil) has no effect on Alzheimer’s disease [3]. Nevertheless, the neuroprotective roles of vitamin E, especially those of tocotrienols, have been well documented in many in vivo and in vitro studies [31,32,33,34,35,36,37].

The primary function of tocochromanols is to protect polyunsaturated lipids against peroxidation. Non-enzymatic lipid peroxidation leads to the destruction of the membrane bilayer built of PUFAs. Thus, peroxidation causes disturbances in cell structure and functionality [13,38], followed by the initiation or/and propagation of various chronic diseases [39,40,41]. It has been shown that Tts display higher bioactivity than Tocs [42,43] because of the presence of double bonds in the isoprenoid side chain. These features make Tt more able to interact with free radicals and may contribute to the better recycling of the molecule to its active reduced form [42,43]. The literature data on the inhibition of lipid peroxidation by Tts as compared to Tocs, especially those including all the homologues, in model systems are innumerous and their results divergent. For example, it was reported that α-Tt possessed significantly higher antioxidant activity than α-Toc in rat liver microsomes [42] and in liposomal membranes [43]. On the other hand, Tts and Tocs did not show significant differences in the antioxidant activities in a homogenous system and in lipoproteins [44]. In that study, both α-tocochromanols were more active than the corresponding γ forms. Moreover, it was shown [45] that the corresponding Tocs and Tts exerted the same antioxidant activities against lipid peroxidation in solution and liposomal membranes and their activity decreased in the order α > β = γ > δ.

In this study, we compared the antioxidant activity of tocochromanols in in vitro models. First, we used plant lipid liposomes as a plant membrane model to study the inhibition of lipid peroxidation by tocochromanols. Lipid peroxidation was generated inside and outside the membrane using two azo-initiators: AMVN (2,2-azobis(2,4-dimethylvaleronitrile)) and AIPH (2,2′-azobis[2-(2-imidazolin-2-yl)propane] dihydrochloride) [46]. We used AMVN, which is hydrophobic, and AIPH, which is hydrophilic; thus, these two initiators localize inside and outside of the membrane, respectively [19], and their homolysis results in the release of free radicals in different membrane compartments. Moreover, using DPPH assay we compared the antioxidant activity of the compounds undergoing analysis in different nanocarriers, such as liposomes, micelles, mixed micelles, and niosomes.

Since one of the causes of many neurodegenerative diseases is supposed to be oxidative damage to neuronal cells, the application of neuronal cell lines and hydrogen peroxide (H_2_O_2_)-induced oxidative stress is frequently used as a model system [47,48,49]. Therefore, to examine the neuroprotective effects of the tocochromanols under investigation on H_2_O_2_-induced cell damage, we used the human neuroblastoma SH-SY5Y cell line differentiated by retinoic acid (RA-SH-SY5Y).

## 2. Results

### 2.1. Inhibition of Lipid Peroxidation in Liposomes Using Lipid-Soluble AMVN

**Figure 2:**
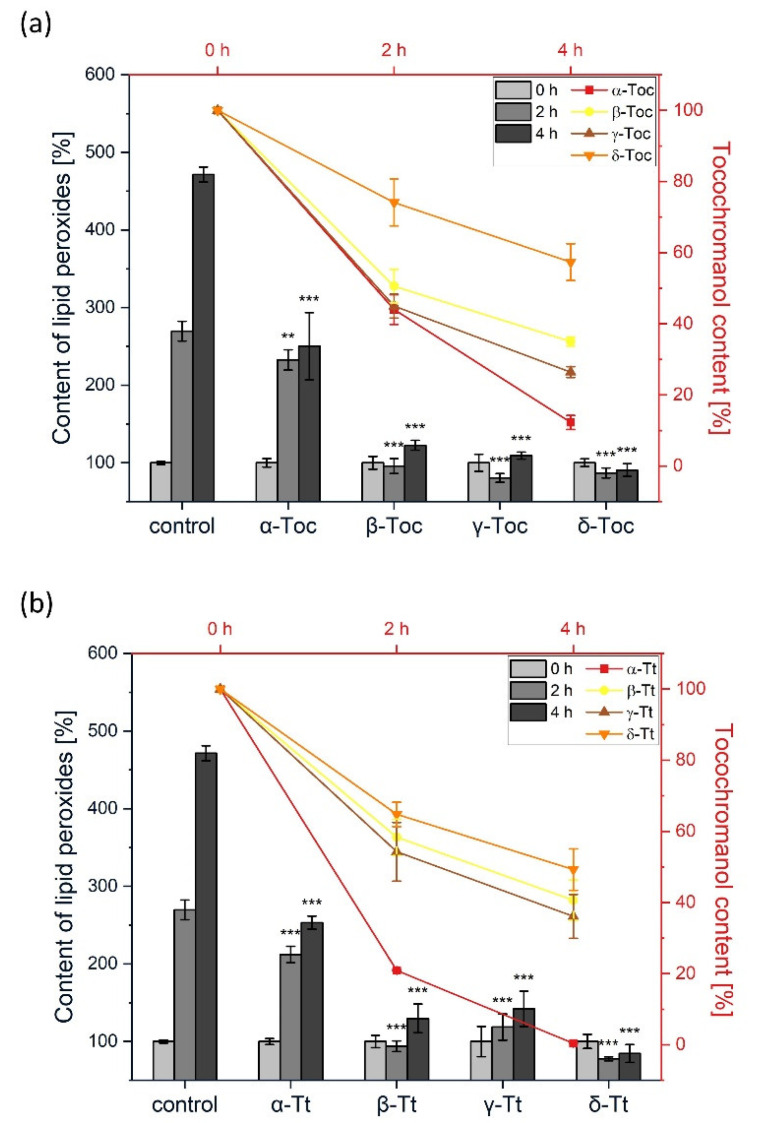
(**a**) The effect of Tocs on the rate of the formation of lipid peroxide in plant lipid liposomes initiated by AMVN (*bars*). Time-course of Tocs level during the peroxidation reaction *(lines*). The reaction was performed at 37 °C. (**b**) The effect of Tts on the rate of the formation of lipid peroxides in plant lipid liposomes initiated by AMVN (*bars*). Time-course of Tts decay during the peroxidation reaction (*lines*). The reactions were conducted at 37 °C. The data are means ± SEM (*n* = 3). Asterisks indicate statistically significant differences compared with control at corresponding time point (** *p* < 0.01, *** *p* < 0.001; one-way ANOVA followed by Duncan’s post hoc test).

Lipid peroxidation in liposomes was initiated both in the hydrophobic and hydrophilic phases. For this experiment, we used liposomes composed of plant-specific glycolipids: monogalactosyldiacylglycerol (MGDG), digalactosyldiacylglycerol (DGDG), sulfoquinovosyldiacylglycerol (SQDG), and phosphatidylglycerol (PG). Using HPLC, we monitored simultaneously the time-course changes of lipid peroxides and levels of tocochromanols. Among Tocs, α-Toc was the least active in the inhibition of AMVN-induced lipid peroxidation, but all the other homologues inhibited the peroxidation reaction almost completely (Figure 2). During the reaction, the α-Toc content showed a rapid decrease after 2 h, reaching 40% of the initial amount, and after 4 h its content decreased to 10% of the initial level (Figure 2a). Under these conditions, β- and γ-Tocs showed similar effects, both in the inhibition of the formation of lipid peroxides, and their consumption, which was approx. 70% after 4 h. During the reaction, δ-Toc consumption was the least pronounced (30% after 2 h, 45% after 4 h). δ-Toc inhibited the peroxidation reaction completely, even after 4 h (Figure 2a).

Figure 2b shows the effect of Tts on lipid peroxidation in liposomes during the reaction initiated by AMVN. The effect of α-Tt was similar to that of α-Toc. As compared to β- and γ-Tocs, the corresponding Tts were slightly less active in the inhibition of lipid peroxidation (Figure 2b). On the other hand, the most evident inhibitory effect was observed for δ-Tt. In our experiment, its level decreased most slowly (c. 50% after 4 h) (Figure 2b). 

**Figure 3:**
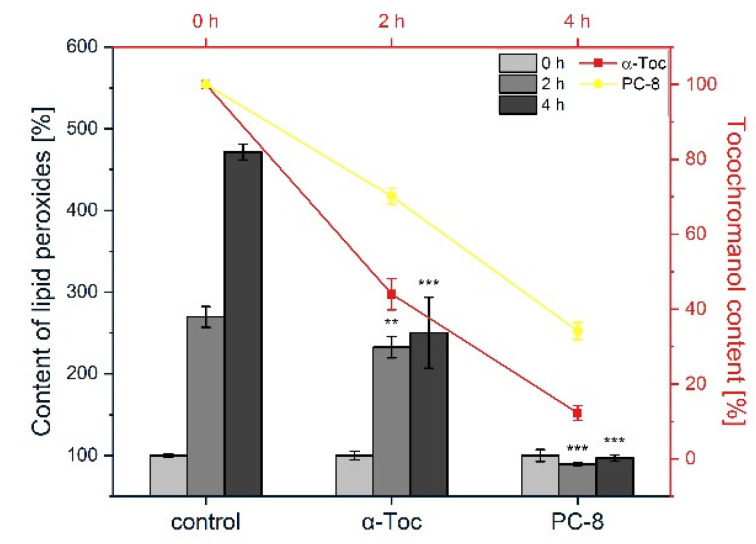
The effect of PC-8 on the rate of the formation of lipid peroxides in plant lipid liposomes initiated by AMVN (*bars*). Time-course of PC-8 decay during the peroxidation reaction (*lines*). The reactions were conducted at 37 °C. The data are means ± SEM (*n* = 3). Asterisks indicate statistically significant differences compared with control at corresponding time point (** *p* < 0.01, *** *p* < 0.001; one-way ANOVA followed by Duncan’s post hoc test).

In liposomes containing PC-8, the peroxidation was completely inhibited (Figure 3). The PC-8 content showed a gradual decrease with time and after 4 h its content reached c. 30% of the initial value (Figure 3). Inhibition of lipid peroxidation in plant lipid liposomes by PC-8 was observed earlier [19], but its activity was similar to that of tocopherols. Our results showed that PC-8 has much better antioxidant properties than α-Toc, and is similar to other tocopherols in the case of AMVN-induced lipid peroxidation. PC-8 was also a better antioxidant as compared to α-, β-, and γ-Tt (Figure 2b and Figure 3). During antioxidant action, PC-8 forms many different oxidation products; among them, several play effective antioxidant roles, e.g., hydroxyl-plastochromanol (PC-OH) [50,51]. This could explain the 70% loss of its content and effective inhibition of lipid peroxidation.

### 2.2. Inhibition of Lipid Peroxidation in Liposomes Using Water-Soluble AIPH

**Figure 4:**
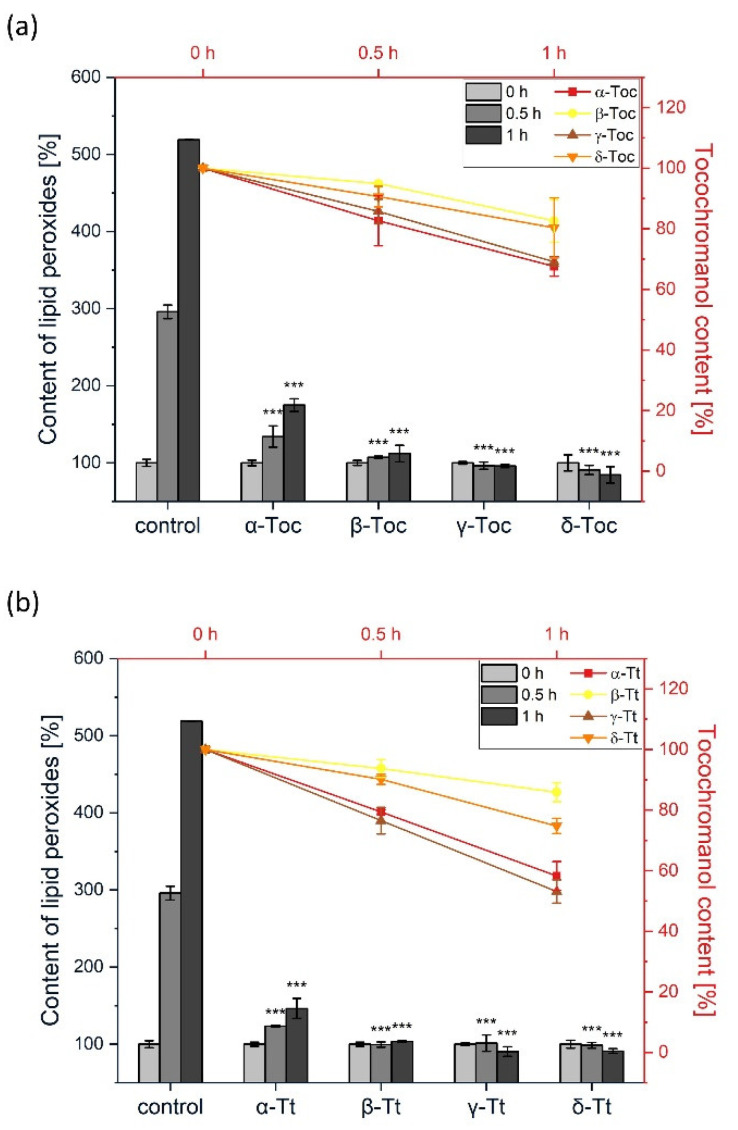
(**a**) The effect of Toc on the rate of the formation of lipid peroxides in plant lipid liposomes initiated by AIPH (*bars*). Time-course of Toc decay during the peroxidation reaction (*lines*). (**b**) The effect of Tt on the rate of the formation of lipid peroxides in plant lipid liposomes initiated by AIPH (*bars*). Time-course of tocotrienol decay during the peroxidation reaction (*lines*). The reactions were conducted at 37 °C. The data are means ± SEM (*n* = 3). Asterisks indicate statistically significant differences compared with control at corresponding time point (*** *p* < 0.001; one-way ANOVA followed by Duncan’s post hoc test).

Lipid peroxidation in liposomes was initiated in the water phase by AIPH. We chose this azo-compound instead of the commonly used AAPH [19,52] because under the same conditions AIPH gives 3.8 times as many free radicals [46]. This azo-initiator has been not applied yet for the study of the antioxidant action of tocopherols or plastochromanol. In our experimental set-up, under the same concentration, AAPH did not oxidize the lipids in a control sample (*data not shown*). The results of the inhibition of AIPH-initiated lipid peroxidation by Tocs are shown in Figure 4. Rather like the effect observed for liposomes containing α-homologues in AMVN-dependent lipid peroxidation, here we also observed a time-course increase in lipid peroxides and the decay of α-forms. During the reaction, the α-Toc content decreased by 35% after 1 h, while that of α-Tt decreased by more than 40% (Figure 4a,b). The formation of lipid peroxides in liposomes containing the other Tocs and Tts (β-, γ-, δ-) was totally inhibited. The decline in β- and δ-Tocs content was 20% after 1 h, whereas the content of γ-Toc was the same as for the α-homologue (Figure 4a). During the AIPH-initiated peroxidation, the content of all the unsaturated tocochromanols decreased gradually over the reaction time.

**Figure 5:**
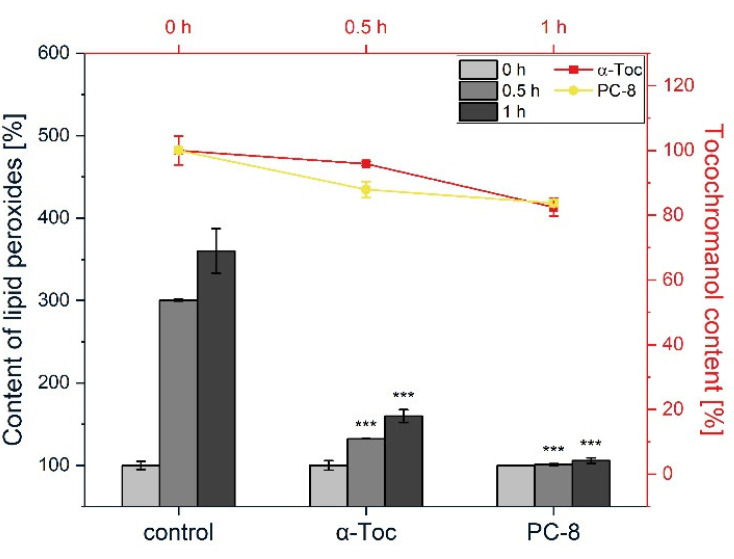
The effect of PC-8 on the rate of the formation of lipid peroxides in plant lipid liposomes initiated by AIPH (*bars*). Time-course of PC-8 decay during the peroxidation reaction (*lines*). The reactions were conducted at 37 °C. The data are means ± SEM (*n* = 3). Asterisks indicate statistically significant differences compared with control at corresponding time point (*** *p* < 0.001; one-way ANOVA followed by Duncan’s post hoc test).

We performed an additional experiment, where the inhibition of the lipid peroxidation activity of α-Toc and PC-8 was compared (Figure 5). In the case of PC-8, we observed inhibition of the formation of lipid peroxides, along with a decrease in its content (20% of the initial value after 1 h) (Figure 5), rather like the case of AMVN-initiated peroxidation. We also compared the antioxidant activity of α-TP. In the reaction mediated by AIPH in liposomes, the level of lipid peroxides was the same as that for α-Toc and α-TP (*data not shown*). Taken together, the results indicate that the chromanol head group of the compounds investigated is positioned close to the membrane interface, which facilitates their antioxidant action in AIPH-mediated lipid peroxidation.

### 2.3. Dynamic Light Scattering Analysis of Tocochromanols Containing Liposomes

**Figure 6:**
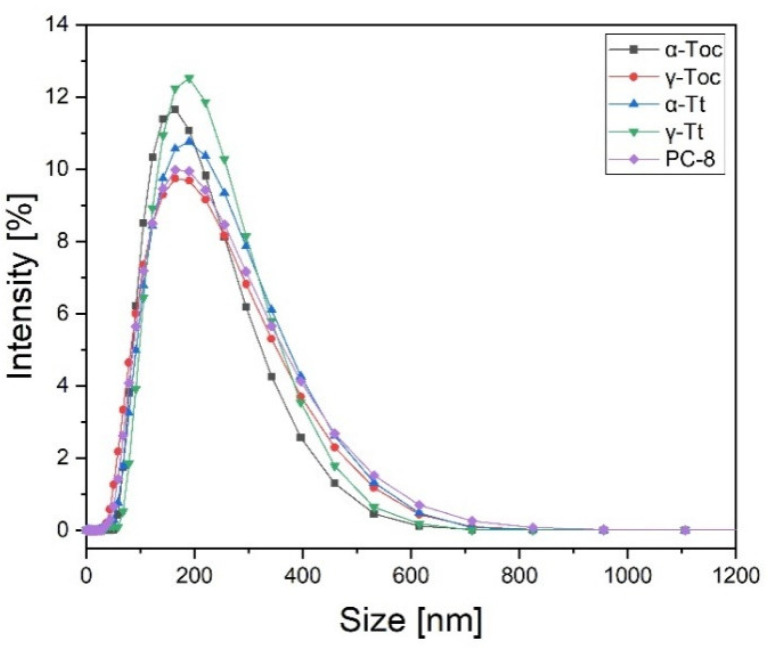
Intensity-based size distribution of the tocochromanol-loaded liposomes (averages of 5 measurements).

The intensity-based size distribution of tocochromanol-loaded liposomes (Figure 6) shows a well-defined peak with a mean of 200 nm and a polydispersity index (PdI) of 0.16–0.24. The size obtained from intensity distribution is the most reliable because it is a result of direct measurement. Figure 6 shows the average hydrodynamic diameter of plant lipid liposomes containing selected tocochromanols. The DLS analysis indicated that liposomes loaded with saturated tocochromanols (α- and γ-Toc) are slightly smaller than those containing unsaturated compounds: α-Tt, γ-Tt, and PC-8. The average diameter of liposomes with Tocs was under 200 nm (186 nm for α-form and 194 nm for γ-Toc). The diameter of liposomes loaded with Tts and PC-8 was above 200 nm (Figure 6).

### 2.4. Antioxidant Activity of Tocochromanols in Different Nanocarriers—DPPH Assay

**Figure 7:**
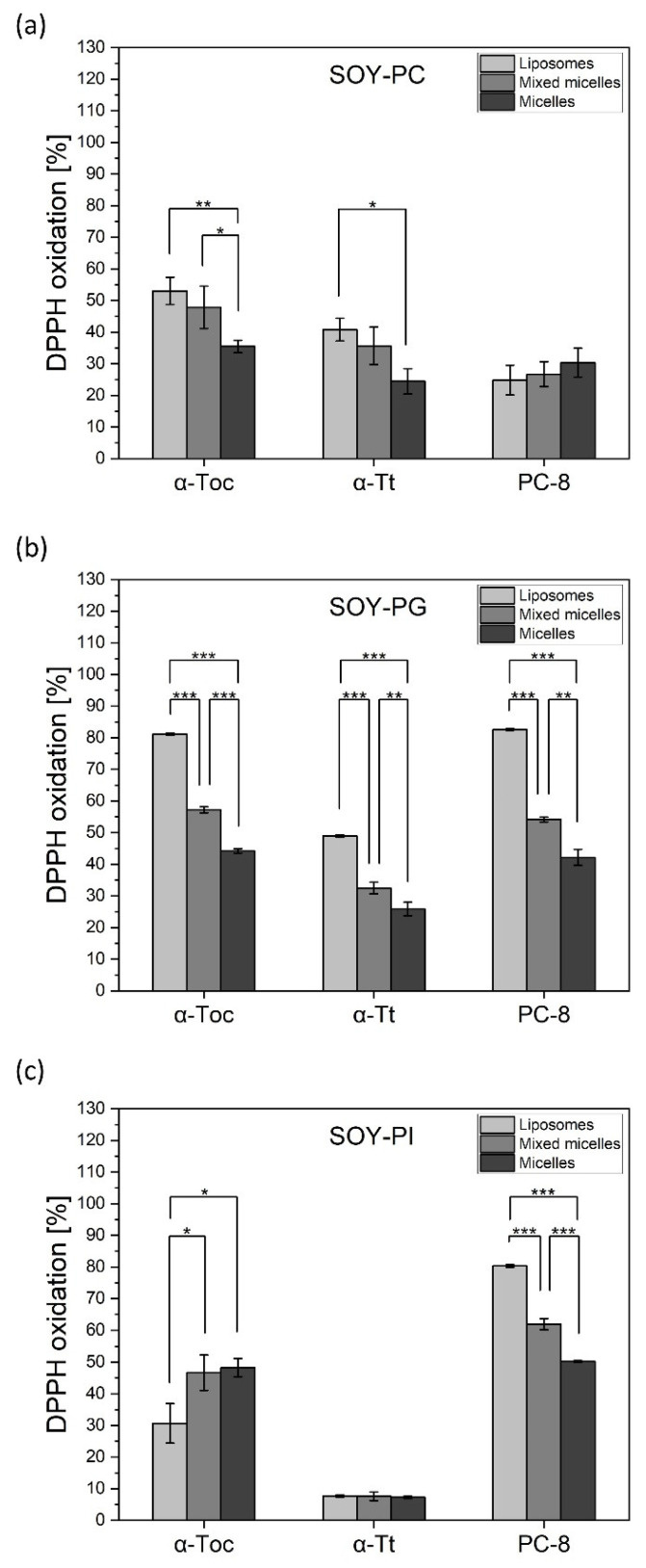
DPPH radical scavenging activity of α-Toc, α-Tt, and PC-8 incorporated into different nanocarriers: liposomes, mixed micelles, and micelles prepared from different soy lipids: (**a**) phosphatidylcholine (lecithin; PC), (**b**) phosphatidylglycerol (PG), and (**c**) phosphatidylinositol (PI) The data are means ± SEM (*n* = 3). Asterisks indicate statistically significant differences (* *p* < 0.05, ** *p* < 0.01, *** *p* < 0.001; one-way ANOVA followed by Tukey’s post hoc test).

**Figure 8:**
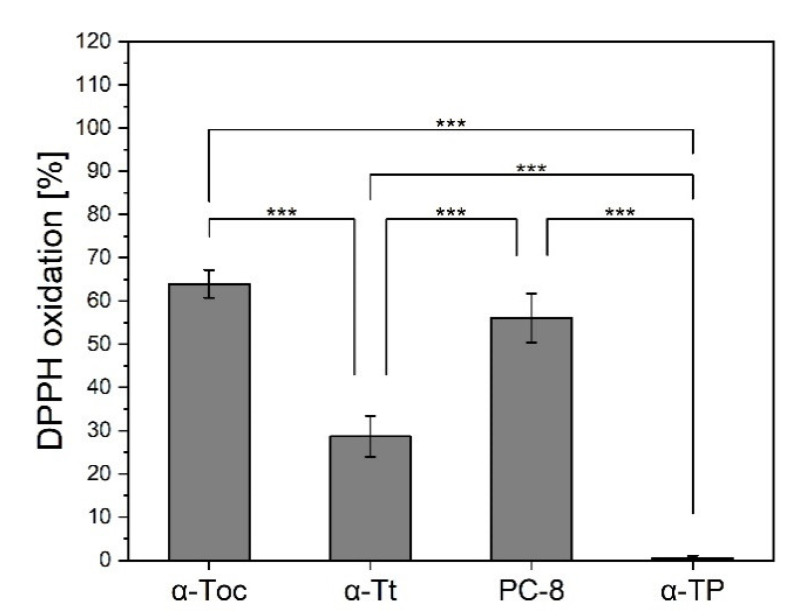
DPPH radical scavenging activity of α-Toc, α-Tt, α-TP, and PC-8 incorporated into niosomes. The data are means ± SEM (*n* = 3). Asterisks indicate statistically significant differences (*** *p* < 0.001; one-way ANOVA followed by Tukey’s post hoc test).

As the comparative antioxidant activity of tocochromanols in different nanocarriers has been not reported so far, in order to compare the antioxidant activity of the selected tocochromanols (α-Toc, α-Tt, γ-Toc, α-TP, and PC-8), depending on different lipid environments, we took measurements of the extent of the oxidation of the DPPH. Tocochromanols were incorporated into different nanocarriers, including (i) liposomes prepared from different soy-originated lipids: phosphatidylcholine (lecithin; PC), phosphatidylglycerol (PG), and phosphatidylinositol (PI); (ii) mixed micelles prepared from a mixture of PG, PC, or PI and 10 mM sodium cholate; (iii) micelles prepared from a mixture of PG, PC, or PI and 30 mM sodium cholate; and (iv) niosomes prepared from Tween 60 and cholesterol in a 1:1 ratio. As shown in Figure 7, tocochromanols displayed different antioxidant activity depending on the lipid used for liposome preparation. Among PC-based nanocarriers, α-Toc and α-Tt displayed the highest antioxidant activity in liposomes (55% and 45% of DPPH oxidation, respectively), while PC-8 showed the opposite effect (~25%). When incorporated into micelles, the activity of tocochromanols was lower (Figure 7a). In PG-nanocarriers, these compounds showed the most pronounced antioxidant activity (80% for α-Toc and PC-8, >50% for α-Tt) among all the lipid systems under investigation (Figure 7b). All the compounds displayed the most pronounced activity in liposomes, followed by mixed micelles and micelles (Figure 7b). The most interesting results were obtained for PI-nanocarriers. α-Toc in PI-liposomes was evidently less effective in DPPH oxidation than in micelles and mixed micelles (Figure 7c), while α-Tt in all the PI-carriers was not active in this respect (less than 10% of DPPH oxidation). PI-liposomes containing PC-8 showed more than 80% oxidation of DPPH, which was 3 times as high as in the case of α-Toc and more than 8 times as high as for α-Tt (Figure 7c). PC-8 antioxidant activity was lower in PI-mixed micelles than in liposomes, but the extent of DPPH oxidation was even higher than that observed for α-Toc. The antioxidant activity of α-TP in liposomes was very low and did not change according to the lipid used (*data not shown*). In order to check the tocochromanol antioxidant activity in nanocarriers, which lack phospholipids, the antioxidants under investigation were incorporated into niosomes. We observed a similar effect for α-Toc and PC-8, while α-Tt activity was more than 2-fold lower (Figure 8). In the niosomal system, α-TP was not active (*data not shown*).

### 2.5. Effect of Tocochromanols against H_2_O_2_-Induced Damage in RA-SH-SY5Y Cells

As the effect of tocochromanols against H_2_O_2_-induced damage in RA-SH-SY5Y cells has been not investigated before, in the present study the neuroprotective potential of the tocochromanols under investigation (0.5–60 µM) after H_2_O_2_ (0.5 mM)-induced RA-SH-SY5Y cells damage was estimated using the LDH release assay and MTT reduction test. Twenty-four hours of treatment with tocochromanols at concentrations up to 60 μM did not bring about any detrimental effect on RA-SH-SY5Y cells. Without the tocochromanols, H_2_O_2_ increased LDH release by c. 150% and reduced cell viability by 30–40%.

**Figure 9:**
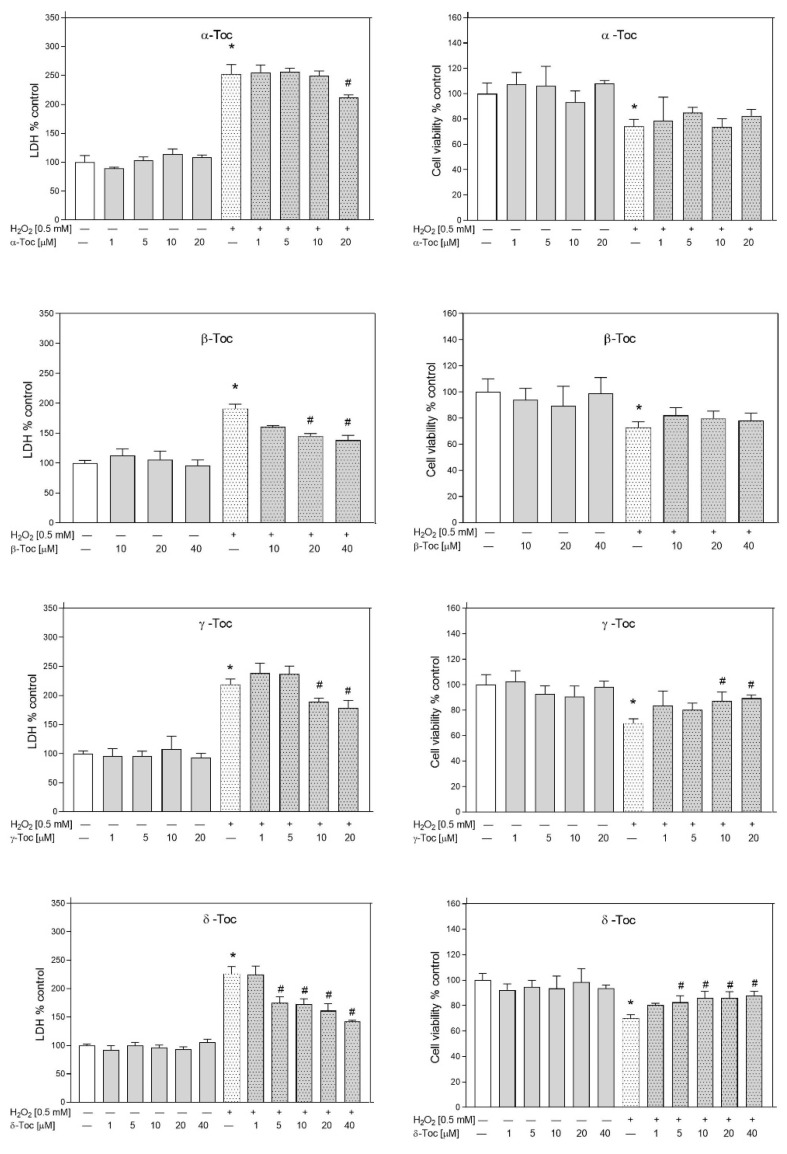
Effect of Tocs against H_2_O_2_-induced damage in RA-SH-SY5Y cells. The cells were pre-treated for 30 min with different ranges of Tocs concentrations (0.5–20 µM) and PC-8 (1–20 µM). Then, 24 h after H_2_O_2_-induced damage (0.5 mM), the cell toxicity by LDH release assay (*left panel*) and cell viability by MTT reduction assay (*right panel*) were measured. Data after normalization to control cells (100%) are presented as the mean ± SEM from 2–4 independent experiments with 5 replicates. All group means were compared by a two-way ANOVA test followed by Duncan’s post hoc test. * *p* < 0.05 versus control culture and # *p* < 0.05 versus H_2_O_2_-treated cells.

Among Tocs, δ-Toc showed the highest protective effect (LDH test—5 μM, *p =* 0.001087; 10 μM, *p =* 0.000906; 20 μM, *p =* 0.000093; 40 μM, *p =* 0.000026; MTT test—5 μM, *p =* 0.02087; 10 μM, *p =* 0.01826; 20 μM, *p =* 0.01608; 40 μM, *p =* 0.01017) followed by γ-Toc (LDH test—10 μM, *p* = 0.04151; 20 μM, *p* = 0.00903; MTT test—10 μM, *p* = 0.020596; 20 μM, *p* = 0.010527) (Figure 9). β-Toc showed a significant inhibitory effect on H_2_O_2_-induced LDH release at two higher concentrations (20 μM, *p* = 0.000242 and 40 µM, *p* = 0.000090) and α-Toc evoked neuroprotective activity only at a concentration of 20 µM (*p* = 0.002458).

**Figure 10:**
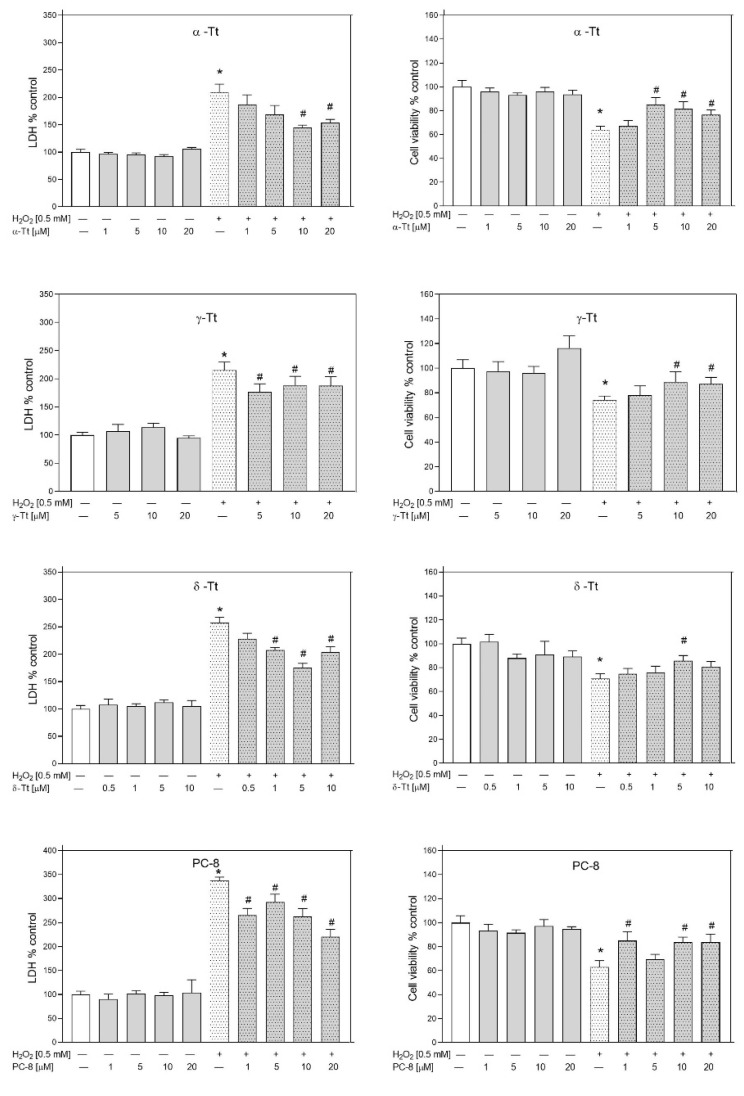
Effect of α, γ, δ-Tt, and PC-8 against H_2_O_2_-induced damage in RA-SH-SY5Y cells. The cells were pre-treated for 30 min with different ranges of Tts concentrations (0.5–20 µM) and PC-8 (1–20 µM). Then, 24 h after H_2_O_2_-induced damage (0.5 mM), the cell toxicity by LDH release assay (*left panel*) and cell viability by MTT reduction assay (*right panel*) were measured. Data after normalization to control cells (100%) are presented as the mean ± SEM from 2–4 independent experiments with five replicates. All group means were compared by a two-way ANOVA test followed by Duncan’s post hoc test. * *p* < 0.05 versus control culture and # *p* < 0.05 versus H_2_O_2_-treated cells.

**Figure 11:**
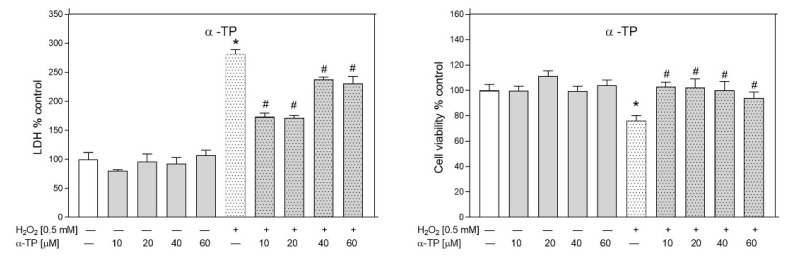
Effect of α-TP against H_2_O_2_-induced RA-SH-SY5Y cells damage. The cells were incubated with α-TP (10–60 µM) and H_2_O_2_ (0.5 mM) for 24 h, followed by measurement of cell toxicity by LDH release assay (*left panel*) and cell viability by MTT reduction assay (*right panel*). Data after normalization to control cells (100%) are presented as the mean ± SEM from 2–4 independent experiments with five replicates. All group means were compared by a two-way ANOVA test followed by Duncan’s post hoc test. * *p* < 0.05 versus control culture and # *p* < 0.05 versus H_2_O_2_-treated cells.

In these tests, Tts were more active as neuroprotectants than Tocs. The strongest inhibitory effect on H_2_O_2_-induced LDH release showed δ-Tt (1 μM, *p* = 0.009720; 5 μM, *p* = 0.000101; 10 μM, *p* = 0.007160), followed by γ-Tt (5 μM, *p* = 0.00431; 10 μM, *p* = 0.03092; 20 μM, *p* = 0.03562) and α-Tt (10 μM, *p* = 0.00028; 20 μM, *p* = 0.00056) (Figure 10). δ-Tt significantly reduced LDH release even at the lowest concentration tested (1 μM). Similarly, PC-8 (1 μM, *p* = 0.000192; 5 μM, *p* = 0.006290; 10 μM, *p* = 0.000162; 20 μM, *p* = 0.000032) showed strong inhibitory effects on H_2_O_2_-induced LDH release. The protective effect of all these compounds (α-Tt—5 μM, *p* = 0.001005; 10 μM, *p* = 0.005042; 20 μM, *p =* 0.038810; γ-Tt—10 μM, *p* = 0.0.04064; 20 μM, *p =* 0.0358; δ-Tt—5 μM, *p* = 0.043505; and PC-8—1 μM, *p* = 0.000738; 10 μM, *p* = 0.000803; 20 μM, *p* = 0.000166) was confirmed by cell viability assay (Figure 10). The water-soluble α-TP showed significant concentration-dependent protective effects, inhibiting H_2_O_2_-induced LDH release (10 μM, *p* = 0.000065; 20 μM, *p* = 0.000046; 40 μM, *p* = 0.033781; 60 μM, *p* = 0.021434) and decrease in cell viability (10 μM, *p* = 0.001053; 20 μM, *p* = 0.001334; 40 μM, *p* = 0.002822; 60 μM, *p* = 0.01189) (Figure 11). The most effective protective activity was found by α-TP at the lowest concentration examined (10 μM).

## 3. Discussion

### 3.1. Free Radical-Initiated Lipid Peroxidation

This is the first comparative study where the antioxidant activity of all the Toc and Tt homologues, along with PC-8, was measured in liposomes. Two azo-initiators, the hydrophobic AMVN and hydrophilic AIPH, were used to examine the sites of the antioxidant action of the tocochromanols. Among all the tocochromanols studied to date, vitamin E (Tocs and Tts) are the lipophilic antioxidants that have been examined the most thoroughly. Our results showed that the corresponding tocochromanol homologues display similar activity in the inhibition of lipid peroxidation generated both in the lipid and water phases. This indicates that the localization of the head group of the tocochromanol within the membrane is crucial for the antioxidant effects. The head group of Tocs is supposed to reside closer to the membrane interface, while that of Tts, which have an unsaturated side chain, are localized deeper in the membrane. The highest inhibitory effect was observed for δ-tocochromanols (Figure 2 and Figure 4), followed by γ- and β-homologues. It has previously been shown that γ-Toc is less active than α-Toc in hydrogen donation, so it reacts less effectively with free radicals [53]. Moreover, hydrogen donation capacity depends on the chromanol group structure [53], which is in agreement with our study, where we observed the following relation in free radical scavenging activity: δ > γ = β > α. It is possible that the oxidation products of β-, γ-, and δ-Toc could also play an antioxidant role, whereas α-tocopherol quinone (α-TQ), the oxidation product of α-Toc, is ineffective against free radicals [19]. Overall, the results suggest that in our experimental setup, Tocs, apart from the α-form, play a significant role in membrane lipids protection. Our results are in agreement with a study conducted previously [54]. The authors showed that individual Tts displayed different antioxidant potency in rat liver microsomal membranes, as well as in RAT-1 immortalized fibroblasts. The most effective homologue was δ-Tt, followed by γ- and α-homologues. This effect was explained by the lowest number of methyl groups among Tts in δ-Tt molecules, which allows them to be easily incorporated into the membrane. Differences in the chemical structure of the tocochromanols under investigation have an impact on the size of the liposomes. Those with unsaturated homologues were slightly larger (Figure 6). In general, the effect of tocochromanols on particle size is small because their shape is very close to cylindrical. A similar effect was observed by Massey and Pownall [55] in model human high-density lipoproteins loaded with α-Toc.

PC-8 differs from the other tocochromanols in terms of its longer prenyl side-chain, which has more double bonds. At the same concentration, the inhibitory effect of PC-8 was considerably more pronounced than that of α-Toc, both in AMVN and AIPH-generated lipid peroxidation (Figure 3 and Figure 5). A study on FeAox-6, a synthetic form of the α-homologue of vitamin E with four conjugated double bonds in the prenyl side-chain, showed even better antioxidant potency than that of all the Tts under investigation [54]. Thus, the higher hydrophobicity of PC-8 could result in a more uniform distribution of its molecules within the cell membrane, as well as higher mobility. Taken together, our results clearly indicate that structural differences in the tocochromanols under examination lead to their different locations in the membrane, as well as to different protection mechanisms of free radical-initiated lipid peroxidation in plant lipid liposomes.

The most unexpected and novel finding of our study is that α-Toc and α-Tt are the poorest antioxidants among the investigated tocochromanols in liposomes when lipid peroxidation is initiated both in the lipid and water phase. Although the majority of the previous literature data indicate α-Toc is the most active among the homologues in the inhibition of lipid peroxidation, it should be realized that the antioxidant activity depends on several factors, such as the model system used, lipid composition of the membrane, antioxidant concentration, and lipid initiator used. It should also be taken into account that under natural conditions, tocochromanol radicals, formed during the antioxidant reaction, are recycled by ascorbate, although the efficiency of this process in vivo is an open question [56].

### 3.2. Antioxidant Activity of Tocochromanols in Nanocarriers

It has been shown many times that the nature of liposomes strongly influences the behavior of incorporated molecules [57,58,59,60]. Depending on the model lipids used, the tocochromanols under investigation displayed different activities. All the antioxidants were more active in liposomes, followed by mixed micelles and micelles (Figure 7). Among the lipids tested, most of the tocochromanols loaded in PG-liposomes were active. Higher antioxidant activity in PC-liposomes was observed for α-homologues, whereas PI-liposomes containing PC-8 were much more active than the other tocochromanols examined. There are many reports showing the effect of tocopherols in pure lipid systems [19,46,61]. It is clear that tocochromanols exert serious effects on lipid membrane properties, such as phase behavior and lipid dynamics. In general, the presence of α-Toc in phospholipid bilayers decreases the motional freedom of the lipid fatty acyl chains in the liquid-crystalline state, which is the predominant state of lipids in biological membranes under physiological conditions. On the other hand, in membranes made from non-bilayer lipids, such as phosphatidylethanolamine, non-bilayer structures such as hexagonal II phases are promoted by the presence of α-Toc [62]. The general features of the location and orientation of α-Toc in phospholipid bilayers are also reproduced in the few atomistic molecular dynamics (MD) simulations. Leng et al. [63] revealed that high disorder increases the probability that a polyunsaturated chain will come into close proximity with the hydroxyl group of the chromanol head group that resides at the surface of the bilayer. Additionally, they observed the fast flip-flop movement of α-Toc, which suggests that the hydroxyl group can also easily penetrate into the interior of a polyunsaturated membrane to intercept lipid peroxyl radicals. This scheme could also be true of the other tocochromanols. Several studies have suggested that α-Toc forms a complex with unsaturated fatty acids, in which a chromanol ring is mainly involved [64,65]. In a complex with linoleic acid, a hydrogen bond links the carboxyl of the fatty acid and the hydroxyl of the Toc molecule, while the double bonds of the fatty acid conform a complementary structure to the methyl groups on the chromanol moiety [66]. Atkinson et al. [67] hypothesized that α-Toc preferentially incorporates into PUFA-rich domains, which are the most susceptible to oxidation. This indicates that the distribution of tocochromanols within biological membranes is not uniform. Taking into account chromanol head group involvement in such a complex formation, the effect of the other homologues would be different. It may be that the stability of the lipid–tocochromanol complex of γ- or δ-form is lower, which facilitates their higher mobility within the bilayer. These kinds of interaction would depend not only on the numbers of methyl groups in the chromanol ring, but also on the type of membrane lipids/phospholipids. 

On the basis of several biophysical techniques, it was found that PI causes packing defects and allows deeper water penetration into the DMPC bilayer [68]. The orientation of the head group of PI in the model membrane has been shown to project perpendicularly from the membrane surface into the aqueous environment, and this allows maximum hydration of the inositol ring [69]. The surface area of this highly hydrated head group is expected to be larger than the cross-section area of the acyl chains, and this might result in looser packing of the acyl chains as compared to the lipids with matching head groups and an acyl chain cross-section area [68]. Altering both hydrophilic and hydrophobic interactions in membranes allows deeper penetration of water, as well as of other biologically active molecules, i.e., proteins [70]. It is worth noting that PI has biological implications in tocopherol metabolism in the human body. Kono et al. [71] showed that interactions with phosphoinositides are also critical for the α-tocopherol transfer protein (α-TTP) function by mediating the binding of α-TTP to PI(3,4)P2 or PI(4,5)P2 in the membrane. Indeed, they observed the direct exchange of PI(4,5)P2 for tocopherol upon incubation of α-TTP with lipid vesicles in vitro. Recently, Chung et al. [72] have shown that the presence of 5-phosphoinositides at the plasma membrane is essential for the α-TTP-mediated transport of α-Toc.

The results of this study suggest that tocochromanol–lipid interactions strongly influence the antioxidant activity of vitamin E and depend on the type of membrane lipids. On the other hand, the presence of the tocochromanols influences the properties of the membrane itself. In this context, tocochromanols could fulfill non-antioxidant functions. As natural components of lipid membranes, they strongly influence the bilayer structure and dynamics and thus can modulate the membrane-dependent processes, such as enzyme activity, signaling cascades, active and passive transport, or gene expression.

### 3.3. Neuroprotective Activity of Tocochromanols

The human SH-SY5Y cell line is a widely used cellular model system for studying the neurotoxic and neuroprotective effects of the compounds under investigation [4,47,48,49]. In these studies, we used the neuronally differentiated SH-SY5Y cells as a screening platform to test the neuroprotective effects of tocochromanols. It should be emphasized that neuroprotective effects of tocochromanols against H_2_O_2_-induced damage in SH-SY5Y cells has been not reported in the literature so far.

Using LDH release and MTT reduction assays, we found that Tts, especially δ-Tt and γ-Tt, were considerably more active than Tocs in the model of oxidative stress-evoked cell damage. Moreover, PC-8 was as active as δ-Tt in inhibiting H_2_O_2_-induced LDH release. Both compounds showed protective effects at the lowest concentration tested (1 μM). The other analogue of Tocs that was investigated, α-TP, effectively inhibited H_2_O_2_-induced detrimental changes in cell viability but at higher concentrations. The neuroprotective activity of the tocochromanols under investigation correlated with their antioxidant activity in liposomes, indicating that the mechanism of protective activity in these compounds consists of protecting cellular membranes against oxidative damage. We are aware of the limitation of our research, which was carried out with only one damaging factor H_2_O_2_. Nevertheless, relying on the available literature, which shows that the protective effect of vitamin E was also observed in many other models, in which the damage was caused by various factors, we can strongly suggest the beneficial potential of vitamin E in the differentiated SH-SY5Y cells. Among others, in the in vitro exposure of murine cerebellar granule cells (CGCs) to neurotoxic damage associated with a variety of metals [73], Tocs and Tts showed a protective effect on CGCs migration against MeHg toxicity. Moreover, in rat PC12 cells, vitamin E (α-Toc) suppressed metal-induced cell damage [74]. Furthermore, the effects of vitamin E (Tts reach fraction and α-Toc) in protecting astrocytes against glutamate injury acted as a potent antioxidant agent in recovering mitochondrial injury due to elevated oxidative stress [75]. The neuroprotective effects of vitamin E, especially those of tocotrienols, have also been well documented in many other in vitro and in vivo studies [31,32,33,34,35,36,37,76]. Thus, based on the available results, it seems legitimate to infer a protective effect of vitamin E expressed in the reduction of the release of LDH induced by H_2_O_2_. Our results indicate a promising application of certain tocochromanols in the pharmacological treatment of neurodegenerative diseases, although this issue requires further study, e.g., in different model systems. In this respect, the application of optimal tocochromanol-carrying structures, such as liposomes, micelles, mixed micelles, and niosomes, might be critical.

## 4. Materials and Methods

### 4.1. Materials

Tocopherol standards of high purity (α-99.9%, β-99.5%, γ-99.5%, δ-99.9%) were purchased from Merck (Darmstadt, Germany), while Tt standards (purity of 99.5%) were obtained from Calbiochem. α-TP was purchased from Sigma Aldrich (Darmstadt, Germany). The PC-8 standard was purified from linseed oil [61]. The chloroplast lipids, namely monogalactosyldiacylglycerol (MGDG), digalactosyldiacylglycerol (DGDG), and sulphoquinovosyldiacylglycerol (SQDG), were obtained from Lipid Products (Surrey, England), while soy-derived lipids, phosphatidylcholine (PC), phosphatidylglycerol (PG), and phosphatidylinositol (PI), came from Avanti Polar Lipids Inc. (Alabaster, Alabama, US). Cholesterol, sodium cholate, and Tween 60 was obtained from Merck Millipore (Sigma Aldrich, (Darmstadt, Germany). 2,2′-Azobis [2-(2-imidazolin-2-yl)propane] dihydrochloride (AIPH) came from TCI Chemicals (Tokyo, Japan), while 2,2′-azobis(2,4-dimethylvaleronitrile) (AMVN) and DPPH were obtained from Sigma Aldrich (Darmstadt, Germany). Solvents used for high-performance liquid chromatography (HPLC) were of HPLC-grade purity and were purchased from Chemland (Stargrad Sz., Poland).

### 4.2. Methods

#### 4.2.1. Lipid Peroxidation of Tocochromanol-Containing Liposomes

Lipid peroxidation was initiated in the water phase by AIPH and in the lipid bilayer by AMVN. In these experiments liposomes were prepared from a mixture of chloroplast lipids MGDG/DGDG/SQDG/PG, 4:2:1:1 (mol/mol), respectively. In those experiments where lipid peroxidation was initiated with AIPH, the stock solution of each tocochromanol in ethanol was mixed with an appropriate volume of 20 mM chloroplast lipids in ethanol and injected into 25 mM HEPES buffer (Sigma Aldrich, Darmstadt, Germany) (pH 6.5), followed by the addition of AIPH in water. The final concentration of tocochromanols and chloroplast lipids was 50 μM and 0.5 mM, respectively, and that of AIPH was 100 μM (for Tocs and Tts) or 250 μM (for PC-8 and α-TP). When lipid peroxidation was initiated by AMVN, a tocochromanol was mixed with ethanol solutions of chloroplast lipids and AMVN. Then, the mixture was injected into 25 mM HEPES buffer (pH 6.5). The final concentration of a tocochromanol and chloroplast lipids was 50 μM and 0.5 mM, respectively, and that of AMVN was 100 μM (for Tocs and Tts) or 250 μM (for PC-8 and α-TP). 

The liposome suspensions were incubated at 37 °C. During incubation, samples were taken at 0, 2, and 4 h in the case of AMVN-initiated lipid peroxidation, and at 0, 0.5, and 1 h in the case of AIPH-initiated lipid peroxidation. Afterwards, 300 μL of each sample was extracted with 600 μL of ethyl acetate via vigorous vortexing in an Eppendorf tube and centrifuged using an Eppendorf mini-centrifuge (2000× *g* × 2 min). Next, 400 µL of the organic upper phase was evaporated under stream nitrogen and dissolved in 130 μL of HPLC eluent. The progress of the peroxidation was followed by taking measurements of the level of lipid peroxides and tocochromanols by HPLC.

#### 4.2.2. HPLC Analysis

The lipid peroxides were analyzed in acetonitrile/methanol/water (72:8:1, *v*/*v*) at a flow rate of 1.5 mL/min and absorbance was detected at 234 nm, using a reverse-phase C18 column (5 µm, 25 cm × 0.4 cm) (MZ-Analysentechnik, Mainz, Germany). The Toc and Tt were analyzed in acetonitrile/methanol/water (72:8:1, *v*/*v*), whereas PC-8 was analyzed in methanol/hexane (340:20, *v*/*v*), all at a flow rate of 1.5 mL/min and with fluorescence detection (λ_ex_ = 290 nm, λ_em_ = 330 nm).

#### 4.2.3. Dynamic Light Scattering (DLS) Measurements

The particle size of the chloroplast lipid liposomes with incorporated tocochromanols was determined using a Malvern Zetasizer Nano S (Malvern Instruments, Worcestershire, UK) particle size analyzer. Each sample was measured five times and the results were expressed as the average value.

#### 4.2.4. Preparation of Tocochromanol-Containing Nanocarriers

In order to prepare liposomes, a stock solution of tocochromanol in ethanol was mixed with ethanol (PC, PG) or an ethanol/chloroform (1:1, *v*/*v*) (PI) solution of soybean lipids. In the case where an ethanol/chloroform solution of PI was used, the solvents were evaporated before the next steps. Then, the appropriate volume of water was added, and the mixture was shaken using a laboratory vortex (Omni Inc., Atlanta, GA, USA) for 2 min, incubated at 37 °C for 30 min, shaken again (2 min), and centrifuged (10,000× *g* × 3 min) using a benchtop centrifuge (Eppendorf, Hamburg, Germany). The final concentration of tocochromanol and lipids was 50 μM and 0.5 mm, respectively.

To prepare mixed micelles and micelles, the procedure described by Zhang and Wang [77] was used, with some modifications. To prepare the micelles, a solution of sodium cholate was added to the suspension of tocochromanol and soybean lipids prepared as described above. The samples were incubated for 30 min at room temperature before the measurements. The final concentration of sodium cholate in the mixed micelles and micelles was 10 mM and 30 mM, respectively.

Niosomes were prepared by mixing a stock solution of a tocochromanol in ethanol with a solution of cholesterol in ethanol/chloroform (1:1, *v*/*v*) and Tween 60 in ethanol. The mixture was incubated at 45 °C for 15 min and evaporated under a stream of nitrogen to obtain the lipid film. Then, the lipid film was vortexed in 1 mL of water for 2 min followed by centrifugation (10,000× *g* × 3 min). The final concentration of cholesterol and Tween 60 was 10 mg/mL and that of tocochromanols was 50 μM.

#### 4.2.5. DPPH Scavenging Assay

The antioxidant activity of the tocochromanol-containing nanocarriers was determined using DPPH scavenging assay. Briefly, 0.5 mL of nanocarrier was mixed with 0.5 mL of ethanol solution of DPPH (0.2 mM) and vortexed for a short time (15 s). Then, the mixture was incubated at room temperature for 30 min in the dark and the absorbance was measured at 529 nm, using a Bio Cary UV-VIS spectrophotometer (Varian Inc., Santa Clara, CA, USA). The DPPH radical scavenging activity was calculated according to the following equation [77]:

(1) $$\mathrm{DPPH}\;\mathrm{oxidation}\;\left(\%\right)=\frac{\left(A_{0}-A_{1}\right)}{A_{0}}\cdot 100\%,$$

where A_0_ is absorbance of the control and A_1_ is absorbance of the sample after 30 min of incubation.

#### 4.2.6. SH-SY5Y Cell Culture and Treatment

Indication of the source of the cell lines: SH-SY5Y cell line from American Type Culture Collection (ATCC), catalog number CRL-2266.

Human neuroblastoma SH-SY5Y cells (ATCC) were grown in Dulbecco’s modified Eagle medium (DMEM) (Gibco) supplemented with a 10% heat-inactivated fetal bovine serum (FBS), 100 units/mL of penicillin, and 100 µg/mL of streptomycin (Sigma, USA, Darmstadt, Germany), and kept in a humidified atmosphere of 5% CO_2_/95% O_2_ at 37 °C as described earlier [78]. After reaching 80% confluence, the cells were seeded onto 96-well plates at a density of 2.5 × 10^4^ cells per well. To obtain differentiated cells (RA-SH-SY5Y), the cells were cultured in a medium supplemented with retinoic acid (RA, 10 µM) for 6 days. On the 7th day, the culture medium was replaced with DMEM containing antibiotics and 1% FBS. The cells were pre-treated for 30 min with the tocochromanols (0.5–60 µM) under investigation followed by 24 h exposure to H_2_O_2_ (0.5 mM). The neuroprotective potential of tocochromanols in the model of oxidative stress-evoked cell damage was quantified using the lactate dehydrogenase (LDH) release assay and MTT reduction test. The effective concentrations of H_2_O_2_ (0.5 mM) were established in our previous study, where this factor caused a reduction in SH-SY5Y cell viability of about 50% (MTT reduction assay) [78]. Tocochromanols stock solution (2 mM) was prepared in ethanol and was stored at −20 °C. The final dilutions of tocochromanols were prepared in a mixture of ethanol and distilled water. The control cells were treated with the same volume of an appropriate vehicle and the solvent was present in cultures at a final concentration of 0.1%.

#### 4.2.7. Cell Viability Assay

Cells viability was quantified using the MTT reduction test based on the enzyme-dependent conversion of a yellow tetrazolium salt to a colored formazan product, the concentration of which is proportional to the number of viable cells. MTT (3-[4,5-dimethylthiazol-2-yl]-2,5-diphenyltetrazolium bromide) was added to each well (at a final concentration of 0.15 mg/mL) 24 h after H_2_O_2_ treatment and incubated for 1 h at 37 °C. The formazan crystals were dissolved with 0.1 N HCl in isopropanol. The absorbance was measured at 570 nm in a 96-well plate-reader (Infinite^®^ M200 PRO, Tecan, Zurich, Switzerland). The data were normalized to the absorbance in the control cells (100%) and expressed as a percentage of the control cells ± SEM established from 2–4 independent experiments with five replicates.

#### 4.2.8. Cell Death Assessment

In order to estimate cell death, the level of lactate dehydrogenase (LDH) released from damaged cells into culture media was quantified as described previously [78]. The intensity of the red color formed in the assay (Cytotoxicity Detection Kit, Roche Basel, Switzerland) was measured at a wavelength of 490 nm and the absorbance of blanks, determined as no-enzyme control, was subtracted from each value. The data were normalized to the amount of LDH released from the control cells (100%) and are presented as the mean ± SEM from 2–4 independent experiments with five replicates.

#### 4.2.9. Statistical Analysis

All results are expressed as the mean ± SEM. The data were analyzed using MS Office Excel 2016 and Origin 8.6 (OriginLab, Northampton, MA, USA). One-way or two-way analysis of variance (ANOVA) with Tukey’s or Duncan’s post hoc tests was used to determine significant differences between means (Statistica 13.3; TIBCO Software Inc., Palo Alto, CA, USA); *p* values below 0.05 were considered statistically significant.

## Data Availability

The data presented in this study are available in article.

## References

[1] Fritsche S., Wang X., Jung C. (2017). Recent Advances in our Understanding of Tocopherol Biosynthesis in Plants: An Overview of Key Genes, Functions, and Breeding of Vitamin E Improved Crops. Antioxidants.

[2] Mene-Saffrane L. (2017). Vitamin E Biosynthesis and Its Regulation in Plants. Antioxidants.

[3] Szymanska R., Nowicka B., Kruk J. (2017). Vitamin E—Occurrence, Biosynthesis by Plants and Functions in Human Nutrition. Mini Rev. Med. Chem..

[4] Lopes F.M., Schröder R., da Frota M.L., Zanotto-Filho A., Müller C.B., Pires A.S., Meurer R.T., Colpo G.D., Gelain D.P., Kapczinski F. (2010). Comparison between proliferative and neuron-like SH-SY5Y cells as an in vitro model for Parkinson disease studies. Brain Res..

[5] Szymanska R., Kruk J. (2018). Novel and rare prenyllipids—Occurrence and biological activity. Plant Physiol. Biochem..

[6] Papas A.M. (1999). CHAPTER Vitamin E: Tocopherols and Tocotrienols. Antioxidant Status, Diet, Nutrition, and Health.

[7] Fiorentino A., Mastellone C., D’Abrosca B., Pacifico S., Scognamiglio M., Cefarelli G., Caputo R., Monaco P. (2009). δ-Tocomonoenol: A new vitamin E from kiwi (*Actinidia chinensis*) fruits. Food Chem..

[8] Niki E., Abe K. (2019). CHAPTER 1 Vitamin E: Structure, Properties and Functions. Vitamin E: Chemistry and Nutritional Benefits.

[9] Kruk J., Pisarski A., Szymanska R. (2011). Novel vitamin E forms in leaves of *Kalanchoe daigremontiana* and *Phaseolus coccineus*. J. Plant Physiol..

[10] Montoya-Arroyo A., Wagner T., Sus N., Müller M., Kröpfl A., Vetter W., Frank J. (2021). Cytotoxicity, cellular uptake, and metabolism to short-chain metabolites of 11′-α-tocomonoenol is similar to RRR-α-tocopherol in HepG2 cells. Free Radic. Biol. Med..

[11] Gee P.T. (2011). Unleashing the untold and misunderstood observations on vitamin E. Genes Nutr..

[12] Yamamoto Y., Maita N., Fujisawa A., Takashima J., Ishii Y., Dunlap W.C. (1999). A new vitamin E (alpha-tocomonoenol) from eggs of the Pacific salmon *Oncorhynchus Keta*. J. Nat. Prod..

[13] Wang X., Quinn P.J. (1999). Vitamin E and its function in membranes. Prog. Lipid Res..

[14] Kruk J., Szymanska R., Cela J., Munne-Bosch S. (2014). Plastochromanol-8: Fifty years of research. Phytochemistry.

[15] Szymanska R., Kruk J. (2010). Plastoquinol is the main prenyllipid synthesized during acclimation to high light conditions in Arabidopsis and is converted to plastochromanol by tocopherol cyclase. Plant Cell Physiol..

[16] Gianello R., Libinaki R., Azzi A., Gavin P.D., Negis Y., Zingg J.M., Holt P., Keah H.H., Griffey A., Smallridge A. (2005). Alpha-tocopheryl phosphate: A novel, natural form of vitamin E. Free Radic. Biol. Med..

[17] Trela A., Szymanska R. (2019). Less widespread plant oils as a good source of vitamin E. Food Chem..

[18] Jiang Q., Elson-Schwab I., Courtemanche C., Ames B.N. (2000). gamma-tocopherol and its major metabolite, in contrast to alpha-tocopherol, inhibit cyclooxygenase activity in macrophages and epithelial cells. Proc. Natl. Acad. Sci. USA.

[19] Nowicka B., Gruszka J., Kruk J. (2013). Function of plastochromanol and other biological prenyllipids in the inhibition of lipid peroxidation-A comparative study in model systems. Biochim. Biophys. Acta.

[20] Myung S.K., Ju W., Cho B., Oh S.W., Park S.M., Koo B.K., Park B.J., Korean Meta-Analysis Study Group (2013). Efficacy of vitamin and antioxidant supplements in prevention of cardiovascular disease: Systematic review and meta-analysis of randomised controlled trials. BMJ.

[21] Wong W.Y., Poudyal H., Ward L.C., Brown L. (2012). Tocotrienols reverse cardiovascular, metabolic and liver changes in high carbohydrate, high fat diet-fed rats. Nutrients.

[22] Husain K., Francois R.A., Yamauchi T., Perez M., Sebti S.M., Malafa M.P. (2011). Vitamin E delta-tocotrienol augments the antitumor activity of gemcitabine and suppresses constitutive NF-kappaB activation in pancreatic cancer. Mol. Cancer Ther..

[23] Peh H.Y., Tan W.S., Liao W., Wong W.S. (2016). Vitamin E therapy beyond cancer: Tocopherol versus tocotrienol. Pharmacol. Ther..

[24] Packer L., Weber S.U., Rimbach G. (2001). Molecular aspects of alpha-tocotrienol antioxidant action and cell signalling. J. Nutr..

[25] Jiang Q. (2014). Natural forms of vitamin E: Metabolism, antioxidant, and anti-inflammatory activities and their role in disease prevention and therapy. Free Radic. Biol. Med..

[26] Devaraj S., Leonard S., Traber M.G., Jialal I. (2008). Gamma-tocopherol supplementation alone and in combination with alpha-tocopherol alters biomarkers of oxidative stress and inflammation in subjects with metabolic syndrome. Free Radic. Biol. Med..

[27] Basambombo L.L., Carmichael P.H., Cote S., Laurin D. (2017). Use of Vitamin E and C Supplements for the Prevention of Cognitive Decline. Ann. Pharm..

[28] de Rijk M.C., Breteler M.M., den Breeijen J.H., Launer L.J., Grobbee D.E., van der Meche F.G., Hofman A. (1997). Dietary antioxidants and Parkinson disease. The Rotterdam Study. Arch. Neurol..

[29] Chang K.H., Cheng M.L., Chiang M.C., Chen C.M. (2018). Lipophilic antioxidants in neurodegenerative diseases. Clin. Chim. Acta.

[30] Yusuf M.A., Sarin N.B. (2007). Antioxidant value addition in human diets: Genetic transformation of Brassica juncea with γ-TMT gene for increased α-tocopherol content. Transgenic Res..

[31] Wolf R., Wolf D., Ruocco V. (1998). Vitamin E: The radical protector. J. Eur. Acad. Derm. Venereol..

[32] Grundman M. (2000). Vitamin E and Alzheimer disease: The basis for additional clinical trials. Am. J. Clin. Nutr..

[33] Takahashi T., Nakaso K., Horikoshi Y., Hanaki T., Yamakawa M., Nakasone M., Kitagawa Y., Koike T., Matsura T. (2016). Rice bran dietary supplementation improves neurological symptoms and loss of Purkinje cells in vitamin E-deficient mice. Yonago Acta Med..

[34] Li Y., Liu L., Barger S.W. (2001). Vitamin E suppression of microglial activation is neuroprotective. J. Neurosci. Res..

[35] Sen C.K., Khanna S., Roy S., Watson R.R., Preedy V.R. (2009). Tocotrienols as natural neuroprotective vitamins. Tocotrienols: Vitamin E beyond Tocopherols.

[36] Ungurianu A., Zanfirescu A., Nitulescu G., Margina D. (2021). Vitamin E beyond its antioxidant label. Antioxidants.

[37] Kumari M., Ramdas P., Radhakrishnan A.K., Kutty M.K., Haleagrahara N. (2021). Tocotrienols ameliorate neurodegeneration and motor deficits in the 6-OHDA-induced rat model of parkinsonism: Behavioural and immunohistochemistry analysis. Nutrients.

[38] Niki E. (2009). Lipid peroxidation: Physiological levels and dual biological effects. Free Radic. Biol. Med..

[39] Maruyama W., Shaomoto-Nagai M., Kato Y., Hisaka S., Osawa T., Naoi M. (2014). Role of lipid peroxide in the neurodegenerative disorders. Subcell. Biochem..

[40] Ramana K.V., Srivastava S., Singhal S.S. (2019). Lipid Peroxidation Products in Human Health and Disease 2019. Oxid Med. Cell Longev..

[41] Shahidi F., de Camargo A.C. (2016). Tocopherols and Tocotrienols in Common and Emerging Dietary Sources: Occurrence, Applications, and Health Benefits. Int. J. Mol. Sci..

[42] Serbinova E., Kagan V., Han D., Packer L. (1991). Free radical recycling and intramembrane mobility in the antioxidant properties of alpha-tocopherol and alpha-tocotrienol. Free Radic. Biol. Med..

[43] Suzuki Y.J., Tsuchiya M., Wassall S.R., Choo Y.M., Govil G., Kagan V.E., Packer L. (1993). Structural and dynamic membrane properties of alpha-tocopherol and alpha-tocotrienol: Implication to the molecular mechanism of their antioxidant potency. Biochemistry.

[44] Suarna C., Hood R.L., Dean R.T., Stocker R. (1993). Comparative antioxidant activity of tocotrienols and other natural lipid-soluble antioxidants in a homogeneous system, and in rat and human lipoproteins. Biochim. Biophys. Acta.

[45] Yoshida Y., Niki E., Noguchi N. (2003). Comparative study on the action of tocopherols and tocotrienols as antioxidant: Chemical and physical effects. Chem. Phys. Lipids.

[46] Yoshida Y., Itoh N., Saito Y., Hayakawa M., Niki E. (2004). Application of water-soluble radical initiator, 2,2′-azobis [2-(2-imidazolin-2-yl)propane] dihydrochloride, to a study of oxidative stress. Free Radic. Res..

[47] Jantas D., Pytel M., Mozrzymas J.W., Leskiewicz M., Regulska M., Antkiewicz-Michaluk L., Lason W. (2008). The attenuating effect of memantine on staurosporine-, salsolinol- and doxorubicin-induced apoptosis in human neuroblastoma SH-SY5Y cells. Neurochem. Int..

[48] Jantas D., Greda A., Golda S., Korostynski M., Grygier B., Roman A., Pilc A., Lason W. (2014). Neuroprotective effects of metabotropic glutamate receptor group II and III activators against MPP(+)-induced cell death in human neuroblastoma SH-SY5Y cells: The impact of cell differentiation state. Neuropharmacology.

[49] Xie H.R., Hu L.S., Li G.Y. (2010). SH-SY5Y human neuroblastoma cell line: In vitro cell model of dopaminergic neurons in Parkinson’s disease. Chin. Med. J..

[50] Szymanska R., Kruk J. (2010). Identification of hydroxy-plastochromanol in Arabidopsis leaves. Acta Biochim. Pol..

[51] Szymanska R., Nowicka B., Kruk J. (2014). Hydroxy-plastochromanol and plastoquinone-C as singlet oxygen products during photo-oxidative stress in Arabidopsis. Plant Cell Environ..

[52] Doba T., Burton G.W., Ingold K.U. (1985). Antioxidant and co-antioxidant activity of vitamin C. The effect of vitamin C, either alone or in the presence of vitamin E or a water-soluble vitamin E analogue, upon the peroxidation of aqueous multilamellar phospholipid liposomes. Biochim. Biophys. Acta.

[53] Huang S.-W., Frankel E.N., German J.B. (1994). Antioxidant activity of alpha- and gamma-tocopherols in bulk oils and in oil-in-water emulsions. J. Agric. Food Chem..

[54] Palozza P., Verdecchia S., Avanzi L., Vertuani S., Serini S., Iannone A., Manfredini S. (2006). Comparative antioxidant activity of tocotrienols and the novel chromanyl-polyisoprenyl molecule FeAox-6 in isolated membranes and intact cells. Mol. Cell Biochem..

[55] Massey J.B., Pownall H.J. (1998). Interaction of alpha-tocopherol with model human high-density lipoproteins. Biophys. J..

[56] Halliwell B., Gutteridge J.M.C. (2007). Free Radicals in Biology and Medicine.

[57] Amiri S., Ghanbarzadeh B., Hamishehkar H., Hosein M., Babazadeh A., Adun P. (2018). Vitamin E loaded nanoliposomes: Effects of gammaoryzanol, polyethylene glycol and lauric acid on physicochemical properties. Colloid Interface Sci. Commun..

[58] Sakdiset P., Okada A., Todo H., Sugibayashi K. (2018). Selection of phospholipids to design liposome preparations with high skin penetration-enhancing effects. J. Drug Deliv. Sci. Technol..

[59] Shishir M.R.I., Karim N., Gowd V., Zheng X., Chen W. (2019). Liposomal delivery of natural product: A promising approach in health research. Trends Food Sci. Technol..

[60] Zhang J., Han J., Ye A., Liu W., Tian M., Lu Y., Wu K., Liu J., Lou M.P. (2019). Influence of phospholipids structure on the physicochemical properties and in vitro digestibility of lactoferrin-loaded liposomes. Food Biophys..

[61] Gruszka J., Pawlak A., Kruk J. (2008). Tocochromanols, plastoquinol, and other biological prenyllipids as singlet oxygen quenchers-determination of singlet oxygen quenching rate constants and oxidation products. Free Radic. Biol. Med..

[62] Hincha D.K. (2008). Effects of alpha-tocopherol (vitamin E) on the stability and lipid dynamics of model membranes mimicking the lipid composition of plant chloroplast membranes. FEBS Lett..

[63] Leng X., Kinnun J.J., Marquardt D., Ghefli M., Kučerka N., Katsaras J., Atkinson J., Harroun T.A., Feller S.E., Wassall S.R. (2015). α-Tocopherol Is Well Designed to Protect Polyunsaturated Phospholipids: MD Simulations. Biophys. J..

[64] Kagan V.E. (1989). Tocopherol stabilizes membrane against phospholipase A, free fatty acids, and lysophospholipids. Ann. N. Y. Acad Sci..

[65] Urano S., Shichita N., Matsuo M. (1988). Interaction of vitamin E and its model compounds with unsaturated fatty acids in homogeneous solution. J. Nutr. Sci. Vitam..

[66] Erin A.N., Skrypin V.V., Kagan V.E. (1985). Formation of alpha-tocopherol complexes with fatty acids. Nature of complexes. Biochim. Biophys. Acta.

[67] Atkinson J., Harroun T., Wassall S.R., Stillwell W., Katsaras J. (2010). The location and behavior of alpha-tocopherol in membranes. Mol. Nutr. Food Res..

[68] Peng A., Pisal D.S., Doty A., Balu-Iyer S.V. (2012). Phosphatidylinositol induces fluid phase formation and packing defects in phosphatidylcholine model membranes. Chem. Phys. Lipids.

[69] Bradshaw J.P., Bushby R.J., Giles C.C., Saunders M.R. (1999). Orientation of the headgroup of phosphatidylinositol in a model biomembrane as determined by neutron diffraction. Biochemistry.

[70] Peng A., Straubinger R.M., Balu-Iyer S.V. (2010). Phosphatidylinositol containing lipidic particles reduces immunogenicity and catabolism of factor VIII in hemophilia a mice. AAPS J..

[71] Kono N., Ohto U., Hiramatsu T., Urabe M., Uchida Y., Satow Y., Arai H. (2013). Impaired alpha-TTP-PIPs interaction underlies familial vitamin E deficiency. Science.

[72] Chung S., Ghelfi M., Atkinson J., Parker R., Qian J., Carlin C., Manor D. (2016). Vtamin E and Phosphoinositides Regulate the Intracellular Localization of the Hepatic alpha-Tocopherol Transfer Protein. J. Biol. Chem..

[73] Shichiri M., Takanezawa Y., Uchida K., Tamai H., Arai H. (2007). Protection of cerebellar granule cells by tocopherols and to-cotrienols against methylmercury toxicity. Brain Res..

[74] Yang L., Shen K., Ji D. (2020). Natural compounds attenuate heavy metal-induced PC12 cell damage. J. Int. Med. Res..

[75] Selvaraju T.R., Khaza’ai H., Vidyadaran S., Abd Mutalib M.S., Vasudevan R. (2014). The neuroprotective effects of tocotrienol rich fraction and alpha tocopherol against glutamate injury in astrocytes. Bosn. J. Basic. Med. Sci..

[76] La Torre M.E., Villano I., Monda M., Messina A., Cibelli G., Valenzano A., Pisanelli D., Panaro M.A., Tartaglia N., Am-brosi A. (2021). Role of Vitamin E and the Orexin System in Neuroprotection. Brain Sci..

[77] Zhang S., Wang X. (2016). Effect of Vesicle-to-Micelle Transition on the Interactions of Phospholipid/Sodium Cholate Mixed Systems with Curcumin in Aqueous Solution. J. Phys. Chem. B.

[78] Szczepanowicz K., Jantas D., Piotrowski M., Staroń J., Leśkiewicz M., Regulska M., Lasoń W., Warszyński P. (2016). Encapsulation of curcumin in polyelectrolyte nanocapsules and their neuroprotective activity. Nanotechnology.

